# Possible Avian Influenza (H5N1) from Migratory Bird, Egypt

**DOI:** 10.3201/eid1307.061222

**Published:** 2007-07

**Authors:** Magdi D. Saad, Lu’ay S. Ahmed, Mohamed A. Gamal-Eldein, Mohamed K. Fouda, Fouda M. Khalil, Samuel L. Yingst, Michael A. Parker, Marshall R. Montevillel

**Affiliations:** * US Naval Medical Research Unit No. 3, Cairo, Egypt; † Ministry of Environment, Cairo, Egypt

**Keywords:** Avian influenza, H5N1, Egypt, migratory bird, common teal, Anas crecca, phylogenetic analysis, letter

**To the Editor:** Wild migratory birds are reservoirs for low pathogenic avian influenza (LPAI) viruses (*1*), but their role in transmitting highly pathogenic avian influenza (HPAI) viruses is hotly debated and unclear (*2*–*4*). Beginning in July 2005, a clade of HPAI (H5N1) viruses rapidly expanded from an apparent focus in western People’s Republic of China and spread to the Middle East, Africa, and Europe (*5*). Genetic analysis of HPAI virus isolates from dead wild birds along major flyways indicated that the strains were closely related to the Qinghai H5N1 A/bar-headed goose/Qinghai/65/2005 virus (clade II) (GenBank accession no. DQ095622). In addition to transmission to domestic poultry, HPAI (H5N1)–infected mute swans have been implicated in direct transmission to humans in Azerbaijan (*6*).

The US Naval Medical Research Unit No. 3 and the Ministry of Environment of Egypt have collaborated since 2003 in obtaining samples from migratory birds to detect circulating influenza viruses. During the 2005–06 migratory birds season, 1,304 migratory birds were sampled from either live bird markets or cage birds trapped by fishermen in Port Said, Damietta, Fayoum, Arish, and Sharm el Sheikh (Appendix Figure, panel A.

A total of 203 cloacal swab samples were positive for influenza A virus matrix gene when tested by real-time PCR, and 2 were also positive for the hemagglutinin 5 (H5) gene by using specific primers (*7*). Of the 2 migratory birds positive for the H5 gene, the first was a common teal (*Anas crecca*) captured in the Nile Delta region of Damietta in October 2005 (Appendix Figure, panel A). Sequencing of the H5 gene showed that this virus was an LPAI most closely related to strain A/mallard/Bavaria/1/2005(H5N2) (GenBank accession no. DQ387854 (*2*).

In January 2006, an influenza A H5 virus (weak positive result) was detected in another common teal (trapped in a cage by a fisherman) sampled from the Damietta region in December 2005 (Appendix Figure, panel A). The low viral load, coupled with the failure to isolate the virus, precluded the laboratory from conducting sequence analysis at the time on the basis of insufficient template material. After the outbreak of influenza A (H5N1) in poultry and humans in Egypt in February 2006, additional retrospective attempts to concentrate RNA were used to assess potential introduction scenarios. After multiple RNA extractions were conducted and the RNA was concentrated, this specimen was found to be positive for the neuraminidase 1 (N1) gene by real-time PCR.

The hemagglutinin gene from both teal strains was sequenced (≈1,596 bp). Sequences were aligned with other influenza A (H5N1) strains from Egypt (9 from humans, 5 from chickens). Twenty other strains with high similarity and from different locations were selected by using a GenBank search algorithm and included in the alignment. A phylogenetic analysis was conducted by using the Kimura 2-parameter model. The LPAI H5 virus strain was used as an outgroup in a neighbor-joining phylogenetic tree. Bootstrap analysis with 500 replicates of sequence data was also conducted by using MEGA 3.1 software (*8*).

Phylogenetic analysis showed clustering of the HPAI (H5N1) strains collected from 1 geographic region (country) (Appendix Figure, panel B). All HPAI (H5N1) strains from Egypt from humans or chickens analyzed clustered with a bootstrap support value of 98%. Furthermore, the A/Teal/Egypt/14051-NAMRU3/2006 (H5N1) strain (collected in December 2005; Appendix Figure, panel A) is an HPAI and is closely related to the parent of the group of viruses isolated in the early 2006 Egypt outbreak, with an average identity of 99.4% with all other strains from Egypt and a bootstrap support value of 96% (Appendix Figure, panel B). Despite the rapid spread of this clade (Qinghai-like strain) to many countries, since late 2005, strains analyzed in this study showed low-level genetic variation (<2%).

Brown et al. reported that species can vary greatly in their response to HPAI (*9*). At least in ducks, it appears that viral shedding is highest in birds with clinical signs of infection, and lowest, as seen in the common teal infected with the HPAI strain in this study, in birds with subclinical infections. These subclinical infections may be due to flock immunity from previous exposure to LPAI H5 virus or genetic factors. This suggestion is conceivable in light of the LPAI H5 virus detected in the other teal a few months earlier.

Such naturally resistant wild birds might serve as vectors for introduction of HPAI viruses into new locations. Data presented herein suggest that an HPAI virus may have been introduced into Egypt through a migratory bird. Whether poultry were infected before mid-February or the teal was infected with influenza A (H5N1) virus by a domesticated species is not unknown. The low degree of viral shedding indicates that detection of any influenza A (H5N1) virus in wild birds in a new region should be immediately followed up with efforts to characterize the virus to control the spread of new subtypes/strains of HPAI into new locations.

## Supplementary Material

Appendix Figure A) Timeline of events of influenza A (H5N1) in migratory birds, domestic poultry, and humans, Egypt, September 2005-May 2006. HPAI, highly pathogenic avian influenza; LPAI, low pathogenic avian influenza. B) Phylogenetic neighbor-joining tree of the hemagglutin gene (1,596 bp) from influenza A virus (H5N1) strains from Egypt and closely related strains from GenBank. GenBank strains are indicated by GenBank numbers. Circles indicate strains from Egypt. Triangle indicates the HPAI (H5N1) teal strain identified in this study. Bootstrap support values (500 replicates) are indicated at each node. Scale bar indicates genetic distance expressed as number of substitutions per site. NAMRU3, Naval Medical Research Unit No. 3.
