# Supplementary figures and images for: Possible Avian Influenza (H5N1) from Migratory Bird, Egypt
Source: Emerg Infect Dis. 2007 Jul;13(7):1120–1. doi: 10.3201/eid1307.061222 (PMC2878221; doi:10.3201/eid1307.061222)

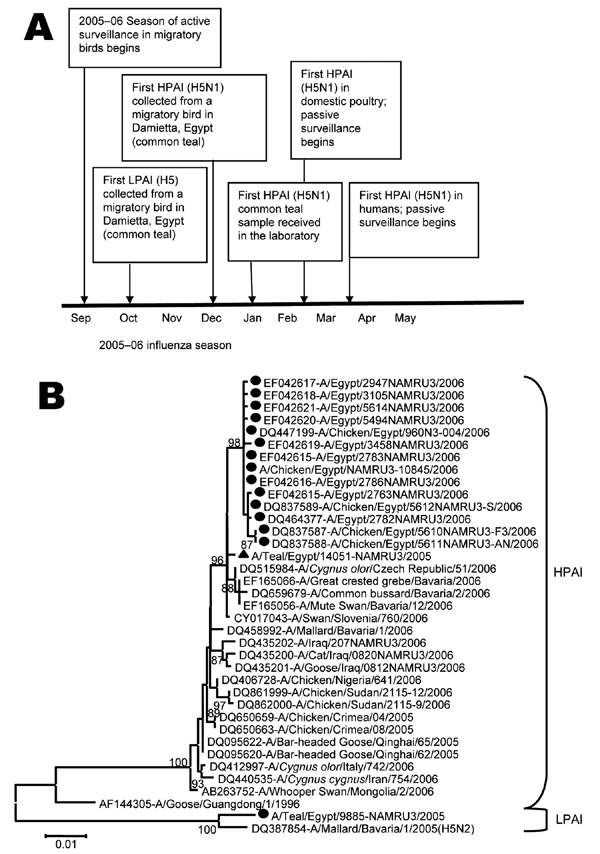

Supplement: Appendix Figure — A) Timeline of events of influenza A (H5N1) in migratory birds, domestic poultry, and humans, Egypt, September 2005-May 2006. HPAI, highly pathogenic avian influenza; LPAI, low pathogenic avian influenza. B) Phylogenetic neighbor-joining tree of the hemagglutin gene (1,596 bp) from influenza A virus (H5N1) strains from Egypt and closely related strains from GenBank. GenBank strains are indicated by GenBank numbers. Circles indicate strains from Egypt. Triangle indicates the HPAI (H5N1) teal strain identified in this study. Bootstrap support values (500 replicates) are indicated at each node. Scale bar indicates genetic distance expressed as number of substitutions per site. NAMRU3, Naval Medical Research Unit No. 3. [file 06-1222_appF-s1.gif]
